# Gut microbiota in the early stage of Crohn’s disease has unique characteristics

**DOI:** 10.1186/s13099-022-00521-0

**Published:** 2022-12-14

**Authors:** Xianzong Ma, Xiaojuan Lu, Wenyu Zhang, Lang Yang, Dezhi Wang, Junfeng Xu, Yan Jia, Xin Wang, Hui Xie, Shu Li, Mingjie Zhang, Yuqi He, Peng Jin, Jianqiu Sheng

**Affiliations:** 1 Medical School of Chinese PLA, Beijing, 100853 China; 2 Department of Gastroenterology, The Seventh Medical Center of Chinese PLA General Hospital, No.5 Nanmencang, Beijing, 100700 China; 3 Capital Medical University, Beijing, 100069 China; 4 Senior Department of Gastroenterology, The First Medical Center of Chinese PLA General Hospital, No.28 Fuxing Road, Beijing, 100853 China

**Keywords:** Crohn’s disease, Early diagnosis, Gut microbiota, 16S rDNA, Dysbacteriosis

## Abstract

**Background:**

Emerging evidence suggests that gut microbiota plays a predominant role in Crohn’s disease (CD). However, the microbiome alterations in the early stage of CD patients still remain unclear. The present study aimed to identify dysbacteriosis in patients with early CD and explore specific gut bacteria related to the progression of CD.

**Methods:**

This study was nested within a longitudinal prospective Chinese CD cohort, and it included 18 early CD patients, 22 advanced CD patients and 30 healthy controls. The microbiota communities were investigated using high-throughput Illumina HiSeq sequencing targeting the V3–V4 region of 16S ribosomal DNA (rDNA) gene. The relationship between the gut microbiota and clinical characteristics of CD was analyzed.

**Results:**

Differential microbiota compositions were observed in CD samples (including early and advanced CD samples) and healthy controls samples. Notably, *Lachnospiracea_incertae_sedis* and *Parabacteroides* were enriched in the early CD patients, *Escherichia/Shigella*,* Enterococcus* and *Proteus* were enriched in the advanced CD patients, and *Roseburia*, *Gemmiger*, *Coprococcus*, *Ruminococcus 2*, *Butyricicoccus*, *Dorea*, *Fusicatenibacter*, *Anaerostipes*, *Clostridium* IV were enriched in the healthy controls [LDA score (log10) > 2]. Furthermore, Kruskal–Wallis Rank sum test results showed that *Blautia*, *Clostridium* IV, *Coprococcus*, *Dorea*, *Fusicatenibacter* continued to significantly decrease in early and advanced CD patients, and *Escherichia/Shigella* and *Proteus* continued to significantly increase compared with healthy controls (P < 0.05). The PICRUSt analysis identified 16 remarkably different metabolic pathways [LDA score (log10) > 2]. Some genera were significantly correlated with various clinical parameters, such as fecal calprotectin, erythrocyte sedimentation rate, C-reactive protein, gland reduce, goblet cells decreased, clinical symptoms (P < 0.05).

**Conclusions:**

Dysbacteriosis occurs in the early stage of CD and is associated with the progression of CD. This data provides a foundation that furthers the understanding of the role of gut microbiota in CD’s pathogenesis.

**Supplementary Information:**

The online version contains supplementary material available at 10.1186/s13099-022-00521-0.

## Background

Crohn’s disease (CD) is a chronic and relapsing gastrointestinal inflammatory disease associated with a high risk of disability [1, 2]. Its incidence has been rapidly increasing worldwide, especially in newly industrialized countries, causing a heavy health economic burden [3]. However, the course of CD is difficult to predict, as it varies from patient to patient. Also, the characteristics of this disease in early stage and the factors influencing the progression of the disease are poorly understood, significantly affecting the clinical decision-making [4]. Further investigations of the influencing factors may provide evidence for CD management, especially in the early treatment, and improves the outcomes of disease [4, 5].

The pathogenesis of CD has not been thoroughly studied. Multiple factors, such as genetics, diet, dysbiosis of gut microbiota and overactivated immune response, are involved in the onset of CD [1, 4, 5]. Among these factors, gut microbiota dysbiosis has been identified as the key factor in the pathogenesis of CD and has been observed in CD patients [6]. Generally, the gut microbiota of healthy adults consists mainly of phyla Firmicutes, Bacteroidetes, and Actinobacteria. In addition, small amounts of Proteobacteria, Verrucomicrobia, Euryarchaeota, and Fusobacteria were found in human fecal samples [7]. Compared to healthy subjects, most studies of CD patients have reported decreased bacterial diversity with an reduction of protective gut microbiota, such as Firmicutes (e.g., butyrate-producing bacteria *Faecalibacterium*, *Roseburia*, *Oscillibacter* and *Coprococcus,* etc.), Actinobacteria (e.g., *Bifidobacterium*) and Verrucomicrobia (e.g., the mucolytic bacteria *Akkermansia muciniphila*), combined with an expansion of putative inflammatory groups, such as *Escherichia coli* (phylum Proteobacteria), *Fusobacterium* spp. (phylum Fusobacteria), and the species *Ruminococcus gnavus* (producer of an inflammatory polysaccharide) [7–10]. As far as we know, many studies have focused on the characteristics of changes in the gut microbiota of CD patients and its predictive or monitoring value in response to CD treatment [11, 12], postoperative recurrence [13, 14], disease activity [15–19], etc. Moreover, studies have also found that the gut microbiota can be used as a biomarker to distinguish CD and non-CD [20]. However, whether the gut microbiota is a cause or a consequence remains unclear, and the relationship between the gut microbiota and the course of CD is still not clearly understood [7, 10, 21]. Perhaps the best way to capture these clues is to investigate the characteristics of gut microbiota changes during the biologic onset or early clinical stage of CD [4].

Although the Paris’s definition of early CD aimed at optimizing CD treatment strategies was proposed in 2012 [22], understanding of the characteristics of gut microbiota changes at this stage is still insufficient, especially in Chinese cohort. More relevant research could help to improve the understanding of CD disease course stage and disease progression and provide evidence for developing early treatment methods targeting the gut microbiota. Therefore, the objective of this study was to analyze the biodiversity of gut microbiota in early-stage and advanced-stage CD patients and healthy controls through high-throughput Illumina HiSeq sequencing targeting the V3–V4 region of 16S ribosomal DNA (rDNA) gene and to compare the differences in the composition of gut microbiota between groups. In addition, we also aimed to identify the dysbacteriosis in the patients with early CD, and find out the specific gut bacteria related to the progression of CD, thus providing new insight for the pathogenesis and early course of CD and prompting the development of clinical prevention, diagnosis and treatment targeting gut microbiota.

## Results

### Patient characteristics and sequencing information

A total of 40 patients with CD were enrolled between December 2018 and October 2021. Groups were matched for age and gender. The mean age of early CD group (EG) was 35.5, and 36.7 years of advanced CD group (AG). Patients with CD but with no CD-related surgical history were all in the active stage [Disease Activity Index of CD (CDAI > 150)]. The detailed clinical features of patients in EG and AG were shown in Table 1.

**Table 1:** Clinical characteristics of subjects [cases (%)]

Clinical index	EG (n = 18)	AG (n = 22)	CG (n = 30)
Age, mean ± SD, years	35.5 ± 13.8	36.7 ± 17.9	35.8 ± 10.5
Gender, male/female	3:1	4:1	3:1
Duration of disease (month)	< 18	> 18	
Clinical symptoms			
Stomachache	13 (65)	16 (80)	
Diarrhea or constipation	5 (25)	9 (45)	
Positive fecal occult blood	2 (10)	3 (15)	
Weight loss	0	2 (10)	
Positive markers for IBD*	7 (35)	13 (65)	
Platelet count (/L, mean)	252*10^9	259*10^9	
FC (ug/g, mean)	621	1096.6	
CRP (mg/dL, mean)	4.1	12.5	
ESR (mm/h, mean)	7.7	16.2	

^*^ASCA or P-ANCA positive

A total of 2,457,406 clean reads were obtained, with an average of 35,614 (29,200–38,965) effective sequences per sample, where the total number of base pairs was 1,051,888,436 bp, and the average sequence length was 414 (400–440) bp. The filtered sequences were clustered at a similarity of 0.97, and the number of OTUs was 9471 with an average of 136 per sample. OTU Venn diagram analysis showed that the OUT numbers of the healthy control groups (CG), EG, and AG were 507, 394, and 459, respectively. Among them, the unique OTU numbers of each group were 81, 11, and 48 (Additional file 1). The Rank abundance curve showed reasonable richness and homogeneity of the species composition of each group (Additional file 1).

### Early CD patients has an altered gut microbiome

#### Early CD patients has unique microbial community characteristics

**Fig. 1:**
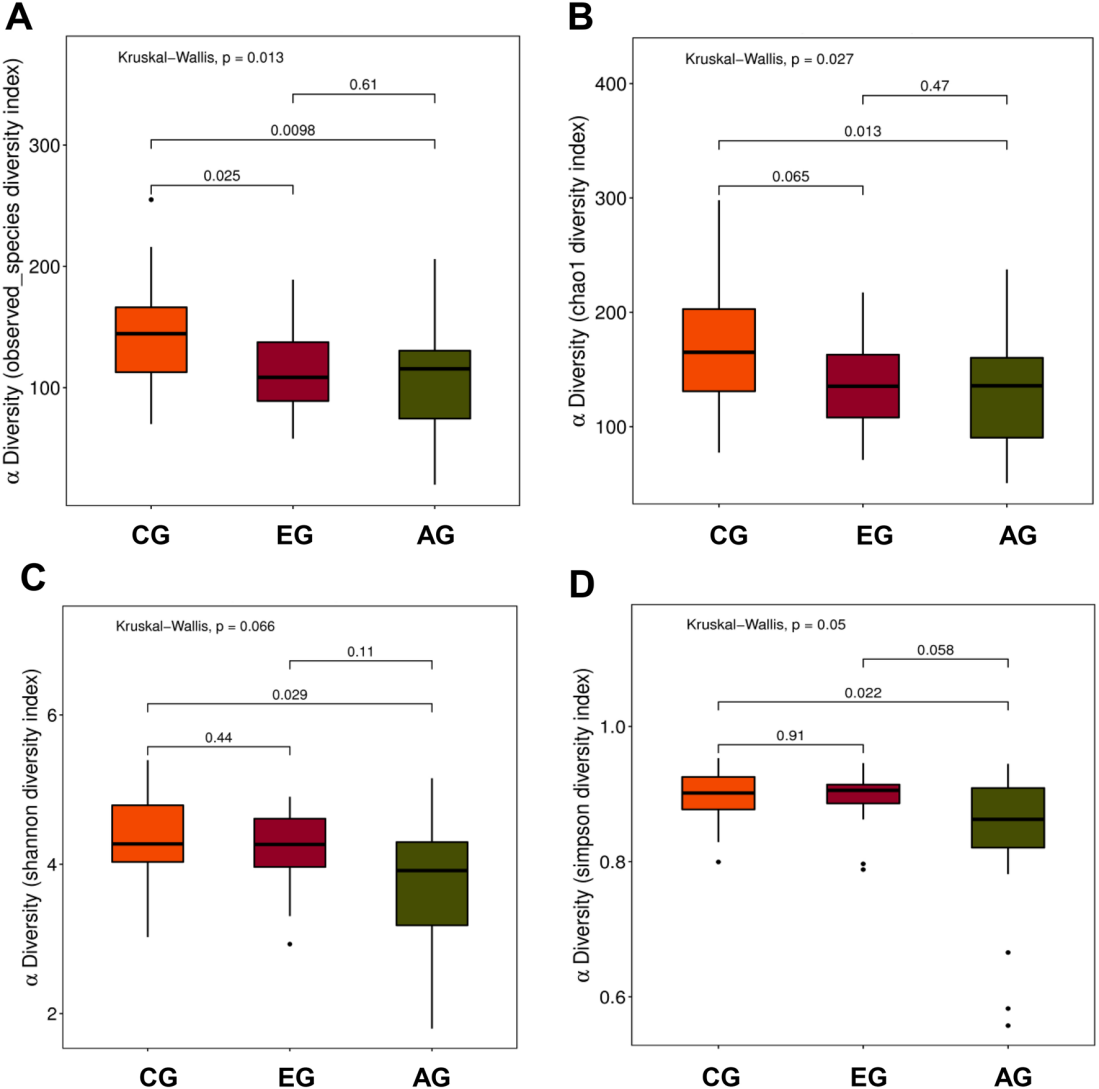
Alpha diversity index box plot. The community richness between early CD groups, advanced CD groups and control groups was showed as **A** (observed species diversity index) and **B** (chao1 index) and the species diversity of microbiota was showed as **C** (Shannon diversity) and **D** (Simpson diversity index). The horizontal axis represents the sample grouping, and the vertical axis represents the Alpha diversity index value of different groups. *CG* control group, *EG* early group of CD patients, *AG* advanced group of CD patients

When comparing bacterial alpha diversity between EG, AG and CG, including community richness (observed species, chao1) and diversity (Shannon and Simpson), we found an overall difference in each diversity index (Fig. 1). Significant differences (P < 0.05) with respect to community richness (observed species) were observed both between EG and CG and between AG and CG. The chao 1 index of was significant differences (P < 0.05) between AG and CG, but the difference was not statistically significant (P > 0.05) between EG and CG. Moreover, the pattern of richness was found to be similar in EG and AG. When considering the species diversity of microbiota (Shannon and Simpson), the differences between AG and CG was statistically significant (P < 0.05). However, the differences between the remaining groups were not statistically significant.

#### Early CD patients has unique microbial community structure

**Fig. 2:**
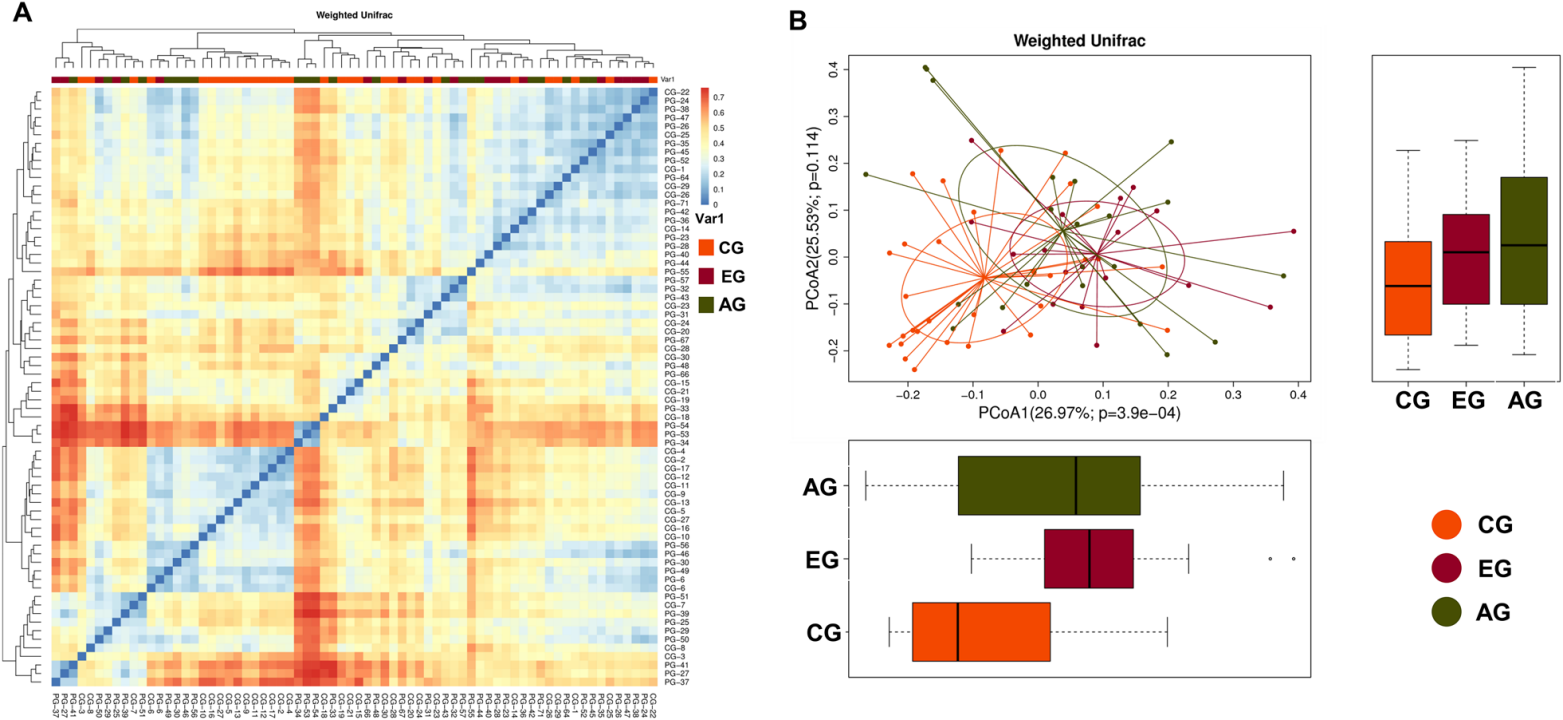
Microbial community structure in early CD groups (EG), advanced CD groups (AG) and control groups (CG). **A** UniFrac heatmap analysis. Patient Group (PG), including EG and AG. And CG means control group. **B** Principal component analysis (PCoA). The percentage represents the contribution rate of the principal dimension to the sample difference

We used principal component analysis (PCoA) to investigate the community structure of microbiota in EG, AG and CG (Fig. 2). We found that samples tended to cluster together based on disease; however, to a certain extent, there was an overlap between all groups (Fig. 2A). CD samples were mostly distinct from those of normal controls, which indicated differences with respect to community structure of the microbiota between CD and controls (Anosim: EG vs CG, R = 0.146, P = 0.008; AG vs CG, R = 0.186, P = 0.001). However, samples of EG and AG were located closely, which suggested a similar bacterial community structure in the context of both early CD and advanced CD (Anosim: R = − 0.033, P = 0.866) (Fig. 2B). Our results indicated that the bacterial community structure in CD was different from that in controls; however, there was no difference with respect to the alterations of bacterial community structure in fecal samples of the early and advanced CD patients.

**Fig. 3:**
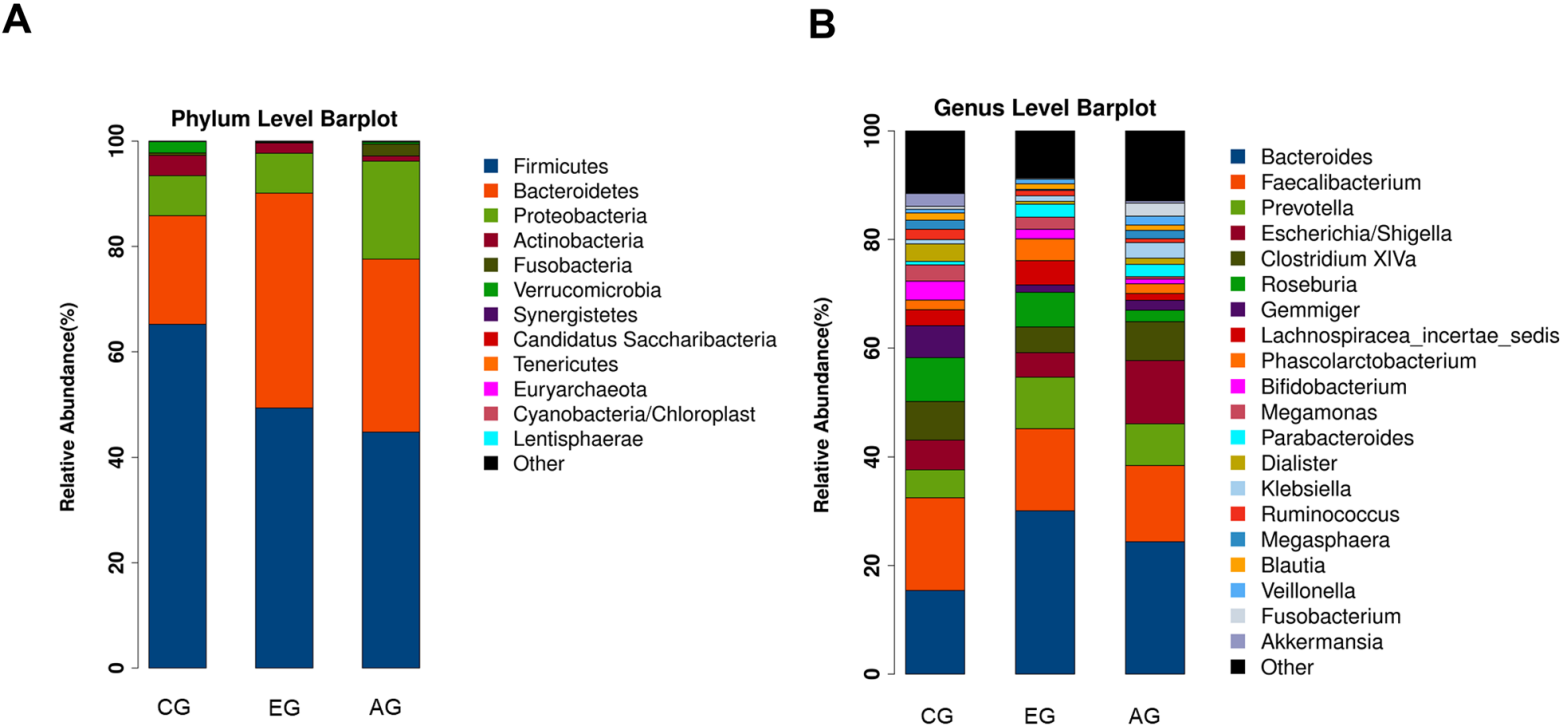
Species classification and abundance analysis of CD patients and controls. **A** The phylum bar graph of the intestinal flora of each group; **B** The genus bar graph of the intestinal flora of each group. *CG* control group, *EG* early group of CD patients, *AG* advanced group of CD patients

#### Species classification and abundance analysis of CD patients and controls

We also analyzed the relative abundance of microbes in the gut microbiome between CD patients and controls at the phylum level and genus level. At the phylum classification level, Firmicutes, Bacteroidetes, Proteobacteria and Actinobacteria accounted for more than 97% of the relative abundance of each group at the phylum classification level. Compared with CG, the abundances of Bacteroidetes increased both in EG and AG, while the abundances of Firmicutes decreased. However, no difference was observed between early and advanced CD at phylum level (Fig. 3A). At the level of genus classification, compared with the controls, the abundance of *Bacteroides* and *Parabacteroides* both increased in EG, while the abundance of *Gemmiger* and *Dialister* both decreased in EG; the abundance of R*oseburia*, *Gemmiger* and *Lachnospiracea_Incertae_sedis* decreased in AG. Compared with the EG, the abundance of *Roseburia* and *Lachnospiracea_Incertae_sedis* in AG both decreased (Fig. 3B).

### Early CD patients harbored unique bacterial biomarkers

**Fig. 4:**
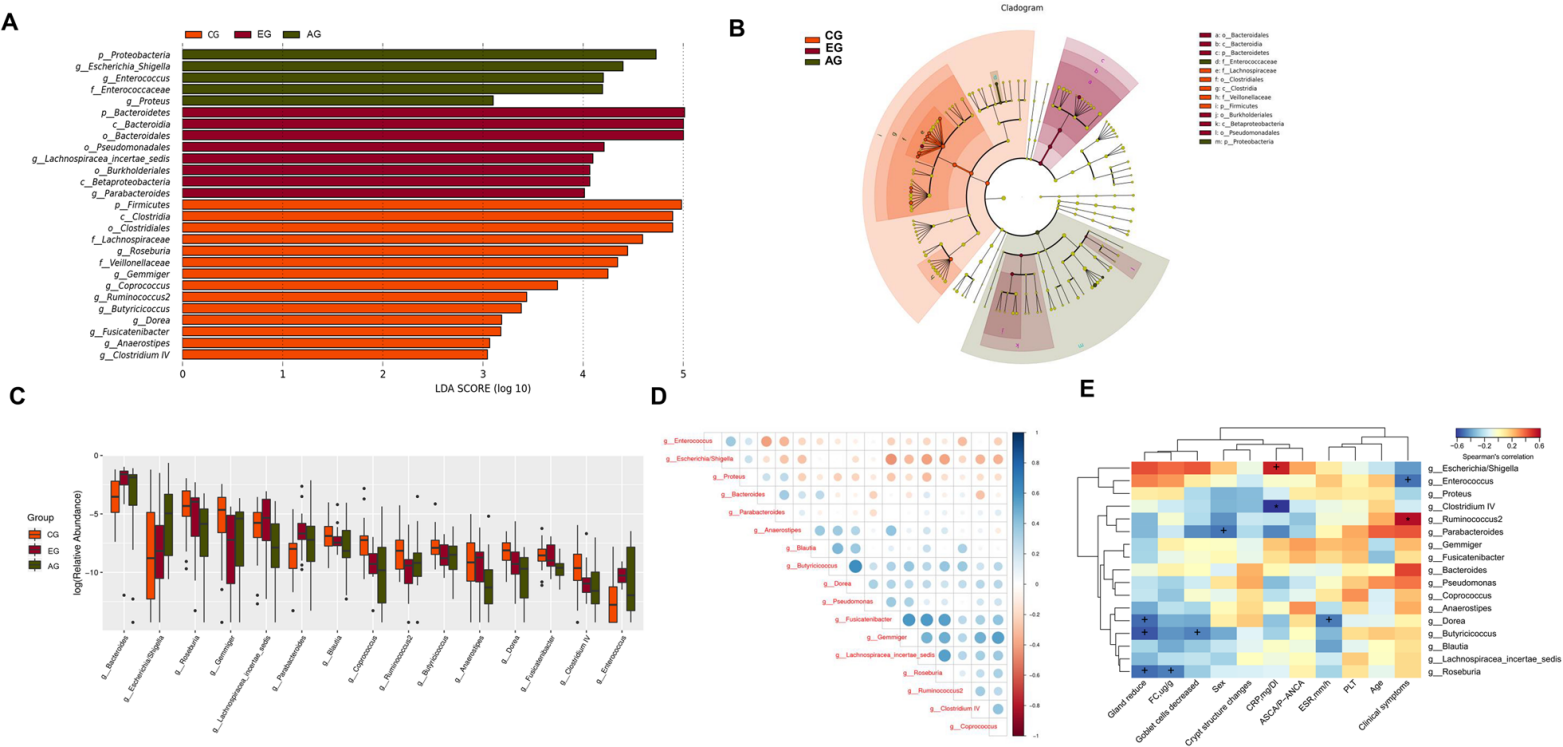
Significant difference analysis of CD patients and controls. **A** and **B** LEfSe analysis. The figure lists bacterial communities with LDA score (log 10) > 3 and P < 0.05. p, phylum; c, class; o, order; f, family; g, genus. **C** Rank sum test analysis between groups (P < 0.05). At the genus level, there are a total of 17 differential bacterial genera, including: *Anaerostipes*, *Bacteroides*, *Blautia*, *Butyricicoccus*, *Clostridium* IV, *Coprococcus*, *Dorea*, *Enterococcus*, *Escherichia/Shigella*,*Fusicatenibacter*, *Gemmiger*, *Lachnospiracea_incertae_sedis*, *Parabacteroides*, *Proteus*, *Pseudomonas*, *Roseburia*, *Ruminococcus 2*. In particular, the abundance sum of *Proteus* and *Pseudomonas* in at least one group is 0, which cannot be shown by Boxplot. **D** Spearman correlation analysis of genera with significant differences among groups. **E** Spearman correlation heat map analysis between clinical markers and characteristic bacteria. *CG* control group, *EG* early group of CD patients, *AG* advanced group of CD patients

Next, we used LEfSe differential analysis to analyze the differences in gut microbial community structure between early and advanced stages of CD. The CG had a high proportion of genera *Roseburia*, *Gemmiger*, *Coprococcus*, *Ruminococcus 2*, *Butyricicoccus*, *Dorea*, *Fusicatenibacter*, *Anaerostipes*, *Clostridium* IV. A high proportion of genera *Lachnospiracea_incertae_sedis* and *Parabacteroides* was observed in EG, and a high proportion of genera *Escherichia/Shigella*, *Enterococcus* and *Proteus* in the advanced stage (Fig. 4A and B, LDA Score (log10) > 2).

We further analyzed the KRUSkal–Wallis Rank sum test and found 17 genera (*Anaerostipes*, *Bacteroides*, *Blautia*, *Butyricicoccus*, *Clostridium IV*, *Coprococcus*, *Dorea*, *Enterococcus*, *Escherichia/Shigella*, *Fusicatenibacter*, *Gemmiger*, *Lachnospiracea_incertae_sedis*, *Parabacteroides*, *Proteus*, *Pseudomonas*, *Roseburia*, *Ruminococcus* 2) with significant differences among different groups (P < 0.05). Compared with CG, the abundance of *Bacteroides*, *Enterococcus*, *Escherichia/Shigella*, *Parabacteroides*, *Proteus* in EG was significantly increased (P < 0.05), while the abundance of *Butyricicoccus*, *Blautia*, *Ruminococcus* 2, *Dorea*, *Anaerostipes*, *Coprococcus*, *Clostridium* IV, *Fusicatenibacter*, *Gemmiger* was significantly decreased (P < 0.05). Compared with the EG, the abundance of *Escherichia/Shigella* and *Proteus* in AG was significantly increased (P < 0.05), while *Roseburia*, *Lachnospiracea_incertae_sedis*, *Fusicatenibacter*, *Coprococcus*, *Blautia*, *Clostridium* IV and *Anaerostipes* were significantly decreased (P < 0.05) (Fig. 4C).

In order to further clarify the dynamic relationship between the symbiotic flora and opportunistic pathogens, the Spearman correlation heat map was used to show the important patterns and relationships among characteristic flora. *Enterococcus* and *Escherichia/Shigella* were positively correlated with each other, while these were negatively correlated with other genus (Fig. 4D). Moreover, we exported the clinical association between the microbiota and CD characteristics [gland reduce, fecal calprotectin (FC), goblet cells decreased, sex, crypt structure changes, C-reactive protein (CRP), anti-saccharomyces cerevisiae antibody (ASCA)/perinuclear antineutrophil cytoplasmic antibody (p-ANCA), erythrocyte sedimentation rate (ESR), platelet, age, clinical symptoms (abdominal pain and diarrhea)] using Spearman correlation heat map analysis, finding a significant negative correlation between *Dorea*, *Butyricicoccus*, *Roseburia* and gland reduce, *Roseburia* and FC, *Butyricicoccus* and goblet cells decreased, *Parabacterodies* and sex, Clostridium IV and CRP, *Dorea* and ESR and a significant positive correlation between *Escherichia/Shigella* and CRP, *Ruminococcus* 2 and clinical symptoms (Fig. 4E).

### KEGG pathways analysis gut microbiome of early CD patients

**Fig. 5:**
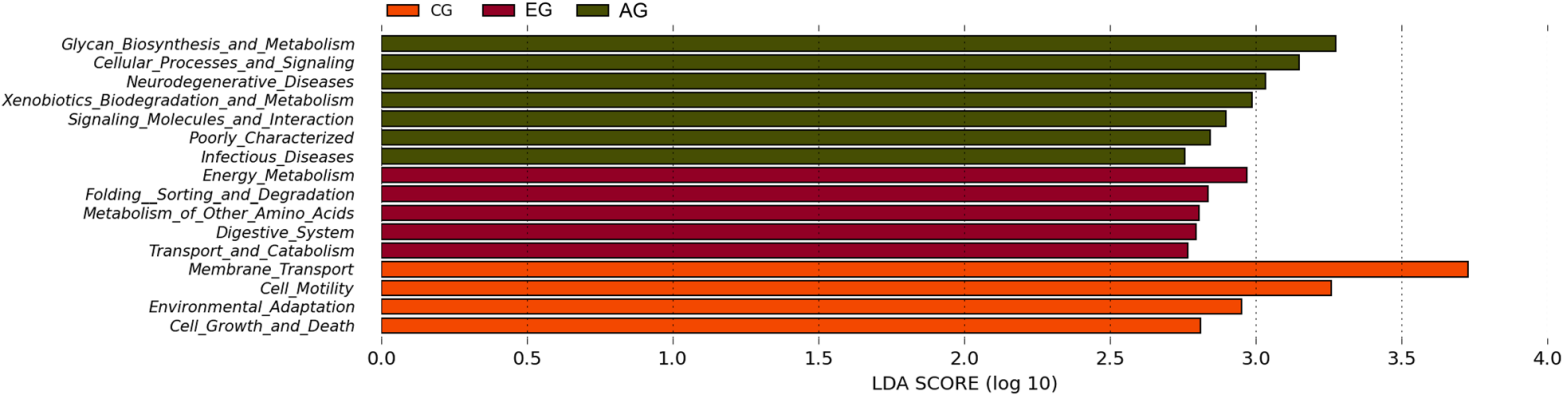
Functional predictions of microbiota present in the fecal of CD patients and healthy controls. Significant KEGG pathways of Level 2 for the microbiome of the CD and healthy groups was identified. *PICRUSt* Phylogenetic Investigation of Communities by Reconstruction of Unobserved States

The phylogenetic investigation of communities by reconstruction of unobserved states (PICRUSt) method [23, 24] was used to predict the KEGG (Kyoto Encyclopedia of Genes and Genomes database) pathways between the microbiome of CD patients and healthy subjects. A total 113 significantly different KEGG orthologues (KOs) were detected in the microbiome of these groups [LDA Score (log10) > 2, P < 0.05, Wilcoxon rank-sum test analysis, data not shown]. Among them, there were 29 KOs enriched in early stage and 19 in advanced stage. In the level 2 of KEGG, 16 of significant pathways were identified. The high functions of EG were related to the pathways of Energy Metabolism, Folding Sorting and Degradation, Metabolism of Other Amino Acids, Digestive System and Transport and Catabolism, while the high functions of microbial genes in CG were related to the pathways of Membrane Transport, Cell Motility, Environmental Adaptation and Cell Growth and Death and the high functions of AG were related to the pathways of Glycan Biosynthesis and Metabolism, Cellular Processes and Signaling, Neurodegenerative Diseases, Xenobiotics Biodegradation and Metabolism, Signaling Molecules and Interaction, Poorly Characterized and Infectious Diseases (Fig. 5). In level 3 of KEGG, 50 of significant pathways were identified (Additional file 1). Notably, the microbial gene function related to Folate biosynthesis, Thiamine metabolism, Lysosome and Peroxisome was characteristically increased in the early CD patients.

## Discussion

The balance between beneficial gut commensals and pathogens is crucial to human health. The dysbiosis of gut microbiota contributes to gut inflammation and may be closely related to the onset and progression of CD [4, 5, 7]. However, our understanding of the relationship between gut microbiota and CD is still relatively poor as it is difficult to detect and diagnose CD in the biologic onset or early stage of disease (e.g., preclinical) [5, 25]. In the present study, we explored the characteristics of gut microbiota changes in the early CD of Paris’s definition and advanced CD [22], and found that significant gut microbiota dysbiosis with unique bacterial biomarkers and metabolic pathway changes in the early stage of Chinese CD patients. The observed dysbiosis was associated with disease progression.

Our study revealed that the dysbiosis in early CD mainly manifested in the following three aspects: first, compared with the health subjects, the fecal microflora community abundance in patients with early CD decreased, however, the observed difference was not statistically significant, while that of patients with advanced CD showed a significant decrease in microbial diversity. Secondly, PCoA results could distinguish the bacterial community structure of patients with early CD from that of the health subjects with statistically significant differences. Finally, the abundance and structure of microbiomes of early and advanced CD were similar. Previous studies have shown that fecal microbiota diversity in western and Chinese patients with CD decreased compared with health subjects [19, 20, 26]. However, the gut microbiota of patients with inactive CD and those with active CD were similar in structure and could not be distinguished by PCoA [26]. Furthermore, altered microbiome composition and stability in CD were not associated with disease activity or long-term course [19]. These inconsistencies may be due to the differences in study design, disease stage, drug use, diet, etc., but the reasons for those conditions are not fully understood, and further research is needed.

We detected detailed compositional alterations in the fecal microbiota of patients with early-stage CD at different taxonomic levels and found a significant reduction in multiple short-chain fatty acid (SCFA)-producing bacteria, including *Blautia*, *Clostridium* IV, *Coprococcus*, *Dorea*, *Fusicatenibacter,* and a significant increase in *Escherichia/Shigella,* and *Proteus*. This trend of gut microbiota imbalance was more obvious in the advanced stage of CD, indicating that gut microbiota imbalance was closely related to the progression of CD inflammation. Although these results were similar to those of previous studies [14, 26–28], these studies do not reflect the role of some key microbiota (e.g., *Parabacteroides*, *Escherichia/Shigella*) in the early course and progression of CD. The present study overcame this deficiency and provides clues for further researches on the mechanism of gut microbiota–host interaction in CD patients.

In the present study, we found that *Bacteroides* increased in CD patients and were mainly enriched in early CD, which could cause opportunistic infections when immune dysfunction or intestinal flora was imbalanced. Previous studies indicated that the changes in the abundance of *Bacteroides* in CD patients were controversial. The increased abundance of *Bacteroides* in the CD group compared with health participates was related to the maintenance of postoperative remission [4, 29]. However, other studies provided that the abundance of *Bacteroides* in CD patients was reduced [27, 30]; *B. thetaiotaomicron*, belonging to the *Bacteroides* was found to prevent weight loss, colonic histopathological changes and the production of inflammatory factors in a mouse enteritis model induced by dextran sodium sulfate (DSS) [31]. Therefore, more research is needed to explore and explain the potential role of *Bacteroides* changes in CD patients, especially in the early stages.

In the present study, *Parabacteroides* were enriched in CD patients, especially patients with early CD. This result was consistent with previous studies, which have shown that *Parabacteroides* had an important role in the pathogenesis of intestinal inflammation, and the decreased abundance of *Parabacteroides* in CD patients was related to the reduction of inflammation [12, 32, 33]. Some studies have shown that certain *Parabacteroides* may aggravate the inflammatory response [34–36]. Oral administration of *P. distasonis* to mice with DSS-induced colitis could significantly aggravate the inflammation [35]. The strain of *Parabacteroides distasonis*, known as CAVFT-HAR46, isolated from microlesions of cavernous fistulas in the intestinal wall of patients with CD, may be the potential pathogenic cause of CD [35]. In addition, *Parabacteroides* had an important role in the pathogenesis of intestinal inflammation; the number of *Parabacteroides* in children with CD under high-level pressure stress was found to increases significantly, suggesting they were related to CD inflammation [36]. However, it remained unclear whether the changes in the abundance of *Parabacteroides* were the cause or the result of intensified or reduced intestinal inflammation.

In addition, our results showed that *Enterococcus* were also enriched in the feces of CD patients, indicating it may be play a key role in the disease course of CD. *Enterococcus*, which can produce extracellular peroxide and damage the DNA of mucosal epithelial cells, is an opportunistic human bacterial pathogen [37]. Previous studies have shown that the abundance of *Enterococcus* in children with CD was significantly increased [38, 39], which was closely related to postoperative recurrence in adult CD patients and was conducive to the fermentation of proteolysis and lactic acid production, while the proteolytic flora was associated with the accumulation of end products known to be toxic to colon cells [40]. In addition, the abundance of *Enterococcus faecalis* in the faeces of CD patients was related to the location of the lesion. Usually, the abundance of *Enterococcus faecalis* in patients with ileal CD is higher than that in patients with ileocolon type [20]. Therefore, we hypothesized that *Enterococcus* may be involved in impairing the intestinal mucosal barrier at the early stage of CD through its secretion or its metabolites, promoting the occurrence of inflammation.

Noteworthy, *Escherichia/Shigella* and *Proteus* were enriched in patients with advanced CD compared with the early patients in the present study, suggesting that these bacteria may be the key factors in the progression of the disease. *Escherichia/Shigella*, a gram-negative bacillus, which could spread between intestinal mucosal cells and eventually colonize, protecting itself from the destruction of innate immunity in the intestine, could cause inflammatory destruction of the intestinal epithelial barrier and lead to the apoptosis of macrophage [20, 41, 42]. Previous studies [20, 27, 43, 44] have also found that *Escherichia* was enriched in CD patients, and some *Escherichia coli* strains with adhesion and invasiveness increased in the ileal mucosa of CD patients, which indicated that *Escherichia* was related to the pathogenesis of CD. Moreover, a cross-sectional study of a large sample showed that *Escherichia* could be used as a landmark to distinguish CD from non-CD [20]. Other studies also found that *Proteus* was related to the severity of colitis in mice [45]. Therefore, *Escherichia/Shigella* and *Proteus* have great significance for understanding the progression and prognosis of CD; however, further research is needed. Our research finding and the above reports indicated that opportunistic pathogens such as *Parabacteroides*, *Bacteroides*, *Enterococcus*, *Escherichia/Shigella* and *Proteus*, which dynamically which changed in the natural course of CD, might play a key role in the occurrence and development of CD. The opportunistic pathogens are expected to become the microbial biomarkers for early CD diagnosis.

The gut microbiota in the early course of CD is characterized not only by the increased opportunistic pathogens but also by the decreased abundance of some common beneficial bacteria, such as SCFA-producing bacteria, which is consistent with the results of previous studies [46]. However, the present study also confirmed that the dynamic reduction of beneficial bacteria in gut microbiota was related to the process of intestinal inflammation and could be used as the potential marker to predict the evolution of CD. The abundance of Firmicutes in early CD patients decreased significantly compared with the controls, mainly due to the significant decrease in genera *Coprococcus*, *Ruminococcus* 2, *Butyricicoccus*, *Dorea*, *Clostridium* IV, etc. This decreasing trend was even more pronounced in patients with advanced CD, especially for *Roseburia*, *Lachnospiracea_incertae_sedis*, *Fusobacterium*, *Blautia*, and *Anaerostipes*, which in advanced CD decreased more significantly compared with patients in early CDs. Previous studies have shown that these bacteria had an important role in maintaining the dynamic balance of intestinal mucosal immune regulation through their metabolites [7, 10, 47]. *Coprococcu*s not only produces butyrate but is also related to the dopamine metabolic pathway, and dopamine is a key brain signal in the pathogenesis of depression, which may explain the higher depression status in CD patients compared to normal people [48]. Moreover, recent studies have pointed to changes in gut microbiota composition and host processing of bacterial-derived metabolites associated with CD, particularly a reduction in the taxa of *Roseburia*, *Lachnospiraceae*, and *Ruminococcus* 2, etc. [49]. We also found the dynamics of characteristic flora using Spearman correlation heat maps, and our results showed that there was some unknown interaction between these characteristic microbiotas, but further studies are needed to confirm this interaction mode of microbiota in order to deeply understand the dynamic changes of intestinal microbiota in CD patients, and to provide a basis for the regulation of intestinal microbiota. Moreover, these characteristic bacteria were correlated with CRP, FC and ESR etc., which reflect the inflammatory state of CD, further suggesting that the gut microbiota dysbiosis may have cascade amplification in CD, thereby promoting the progression of CD and providing clues for exploring the application of existing CD-related biomarkers and intestinal micro-flora in the early diagnosis of CD in future. Taken together, our results further improve insights into the mechanism of gut microbiota in CD, considering that gut microbiota dysbiosis may be more reversible in the early stage of CD than in the progressed patients. Therefore, targeting intestinal microbiota in the early stage of CD may be more meaningful.

Although many bacteria and metabolites associated with CD have been identified, understanding the mechanisms through which microbes influence the occurrence and development of CD requires an extension from association to causation, and the functional analysis of gut microbiota provides a perspective. Functional analysis which has unique significance in the differentiation between groups is often more important than species composition analysis in biological value. PICRUSt (Phylogenetic Investigation of Communities by reconstruction of unobserved States) studies community phyloevolution through recessive state reconstruction, the software predicts metagenomic functional composition based on 16S rDNA and reference sequence databases [23, 24]. By comparing the results of functional analysis of metagenomic sequencing data and corresponding 16S predicted functional analysis, it is found that the accuracy of this method is 84–95%, and the functional analysis of intestinal microbiota and soil microbiota is close to 95%, which can greatly reflect the functional gene composition in the sample [24]. Our PICRUST results suggested that an imbalance in gut microbiota was involved in the progression of CD by changing their gene function, which could provide important reference information for the study of downstream microbiota interaction/response mechanism.

There were some limitations in this study. First, this was a cross-sectional study, but it was nested within a longitudinal prospective Chinese CD cohort [50], which to some extent eliminates possible confounding factors to further understand and validate the dynamic changes of gut microbiota during natural CD processes, and to develop suitable microbial biomarkers for early diagnosis. However, it should be pointed out that the Paris’s definition of early CD is a disease stage defined to optimize treatment strategies [22], which can only partly reflect the early stage of the disease course. Therefore, it is necessary to capture CD patients at an earlier stage or preclinical or even biological stage to truly reveal the natural history of CD through prospective follow-up cohort studies, and establish a biobank to disclose the dynamic changes and roles of gut microbiota in the pathogenesis and disease progression of CD. Second, the sample size of the current study was relatively small, further large-sample multi-omics studies were needed to clarify the potential mechanisms and pathways of gut microbiota imbalance involved in the occurrence and development of CD in order to provide a basis for updating the prevention and treatment strategies of CD. Third, this study only analyzed the fecal flora which was similar to the mucosal associated flora of patients with CD [8]. More tests for analyzing the intestinal mucosal associated flora are needed to further elucidate the role of gut microbiota in the biologic onset or early clinical stage of CD. Furthermore, despite the widespread use of the 16s rDNA sequencing method, it was often challenged that use of different sequence processing pipelines may bring ambiguous results, such as different alignment methods, OTU binning procedures, different kits used to extract DNA, the 16S rDNA regions amplified, and reference databases, etc. [51]. Recently, discussions of standards and pipelines have helped researchers improve the data quality, such as the standard for human fecal sample processing [52], normalization strategies for data characteristics [53] and the future use of metagenomic sequencing as a replacement. In this study, we have used the state-of-the-art pipeline to ensure the reliable interpretation of the 16S sequencing data, and we also look forward to more remarkable finding in our future studies with metagenomic sequencing and integrated multi-omics strategies.

Despite these limitations, the present study demonstrated that the progressive consumption of SCFAs producing bacteria during the course of CD significantly affects the functional metabolism, which is not conducive to the maintenance of intestinal epithelial integrity and the regulation of inflammatory response, and may be a potential factor of etiology in the early stage of CD. In addition, the progressive rise of opportunistic pathogens exacerbates gut dysbiosis during CD, which in turn affects disease progression.

## Conclusions

Our study reported that the gut microbiota of patients with early-stage CD is characterized by a significantly decreased of SCFA-producing bacteria, including *Blautia*, *Clostridium* IV, *Coprococcus*, *Dorea*, *Fusicatenibacter* and a significantly increased of *Escherichia/Shigella* and *Proteus* compared to healthy controls. This trend was particularly evident with the progression of the disease, and *Escherichia/Shigella* may be the key genera in CD disease progression; however, this should be further studied.

## Methods

### Subject recruitment

This cross-sectional study was nested within a longitudinal prospective Chinese CD cohort at the Seventh Medical Center of Chinese PLA General Hospital (Clinical Trials: ChiCTR-1900022728) and was conducted in accordance with the principles of good clinical practice. As reported in our previous study, we achieved early diagnosis of CD by prospectively following up patients with unexplained ileocecal inflammatory lesions for a median of 27 months [50]. These subjects were all from early-diagnosed CD patients (December 2018 to October 2021). The diagnosis of CD is based on standard clinical, endoscopic, radiological and histological criteria [54]. Patients were divided into the early group (EG) and the advanced group (AG) according to the consensus of international experts in Paris [22]. Inclusion criteria included: age 18–75 years; meeting the diagnostic criteria for CD [54]; the patients of EG without complications were all newly diagnosed cases and meet the Paris expert consensus [22]; the prebiotics, probiotics, antibiotics, glucocorticoids, immunosuppressants, biological agents, etc. has not been used in all participates for at least 2 months. Exclusion criteria included: Patients with other immune-related diseases such as ankylosing, spondylitis and psoriasis; Patients with other basic diseases such as diabetes, hypertension and cardiovascular diseases; Patients with suspected of infectious enteritis by stool culture or other etiological examination. Thirty healthy people who received physical examination in our hospital during the same period were included*.*

The study was approved by the Ethics Committee of the Seventh Medical Center of the Chinese PLA General Hospital (#2016-45, #2017-46), and informed consent was obtained from all participates. The sequencing data have uploaded to the GenBank (Bio project ID: PRJNA784251, http://www.ncbi.nlm.nih.gov/bioproject/784251). All authors had access to the study data and have reviewed and approved the manuscript.

### Sample collection and DNA extraction

Fresh fecal samples were collected from the recruited subjects and then transported to the laboratory with an ice pack within 2 h. All samples were frozen immediately and stored at – 80 °C for further analyses. DNA was extracted from fecal samples using based on the Manual of QIAamp Fast DNA Stool Mini Kit (Qiagen, Germany).

### 16S rDNA gene sequencing

The V3–V4 region of the bacteria 16S ribosomal RNA genes was amplified by PCR reaction (95 °C for 3 min, followed by 30 cycles at 98 °C for 20 s, 58 °C for 15 s, and 72 °C for 20 s and a final extension at 72 °C for 5 min) using barcoded primers: 341F 5′-CCTACGGGRSGCAGCAG-3′ and 806R 5′-GGACTACVVGGGTATCTAATC-3′. A total of 30 uL mixture containing 15 uL of 2× KAPA Library Amplification ReadyMix, 1 uL of each primer (10 uM), 50 ng of template DNA, and ddH20 in each sample were performed using PCR reactions.

DNA extraction and amplification were performed on the negative controls consisting of an empty sterile storage tube, using the same procedures and reagents as the fecal sample. There was no detectable amplification in the negative controls. Amplicons were extracted from 2% agarose gels, purified using the AxyPrep DNA GelExtraction Kit (Axygen Biosciences, Union City, CA, U.S.) according to the manufacturer’s instructions and quantified using Qubit 2.0 (Invitrogen, U.S.). All quantified amplicons were pooled to equalize the concentrations for sequencing using Illumina MiSeq/HiSeq (Illumina, Inc., CA, USA). The paired end reads of 250 bp were overlapped on their 3 ends for concatenation into original longer tags by using PANDAseq (https://github.com/neufeld/pandaseq, version 2.9). DNA extraction, library construction and sequencing technology were conducted in Realbio Genomics Institute (Shanghai, China).

### Process of sequencing data

Assembled tags, trimmed of barcodes and primers were further checked to clarify their rest lengths and average base quality. 16S tags were restricted between 220 and 500 bp in order to restrict the average Phred score of bases more than 20(Q20) and less than 3 ambiguous N. The copy number of tags was enumerated and redundancy of repeated tags was removed. Only the tags with frequency > 1, which indicated to be more reliable, was clustered into Operational Taxonomic Units (OTUs). OTUs were clustered with 97% similarity using UPARSE (http://drive5.com/uparse/) and chimeric sequences were identified and removed using Usearch (version 7.0.1090). Each representative tags were assigned to a taxon by RDP Classifier (http://rdp.cme.msu.edu/) according to the RDP database (http://rdp.cme.msu.edu/) using a confidence threshold of 0.8. OTU profiling table and alpha diversity analyses were also achieved by python scripts of QIIME (version 1.9.1).

### Statistical analysis

Data of clinical characteristics are expressed as the mean ± standard deviation (SD). Categorical data is represented by the number of cases and percentage. These analyses were performed using the SPSS 26.0 software (IBM Corporation, Armonk, NY).

Photorefractive curves were created using R software to ensure sufficient sequencing depth for each sample. To assess the significance of differences between groups, the relative abundance of each bacterial group in R-3.2.3 (http://cran.r-project.org) was tested by Chaol index, Simpson index and Shannon index, all used for Alpha diversity. Weighted UniFrac and Principal Co-ordinates Analysis (PCoA) were used to analyze differences in beta diversity. Wilcoxon rank sum test was performed using SPSS 26.0 software to identify the top 20 differentially abundant species in the early CD group and the advanced CD group. The rank sum test was used for continuous variables to analyze factors such as clinical indicators; the χ^2^ test was used for categorical variables; and Fisher’s exact test was used for discrete variables. P < 0.05 was considered statistically significant.

## Supplementary Information

**Additional file 1: Figure S1.** Sequencing information. A. OTUs Venn diagram analysis; B. Rank abundance curve. The X-axis represents the Rank of OTUs Abundance, and the Y-axis represents the corresponding OTUs Abundance. The Rank-Abundance curve can intuitively reflect the classified Abundance and evenness contained in the sample, that is, in the horizontal direction, the higher the value of the curve on the horizontal axis, the higher the Abundance. In the vertical direction, the flatter the curve, the more uniform the species distribution. **Figure S2. **Functional predictions of microbiota present in the fecal of CD patients and healthy controls. Significant KEGG pathways of Level 3 for the microbiome of the CD and healthy groups was identified. PICRUSt, Phylogenetic Investigation of Communities by Reconstruction of Unobserved States.

## Data Availability

The original contributions presented in the study are included in the article/supplementary material, further inquiries can be directed to the corresponding authors.

## Abbreviations

CD: Crohn’s disease
EG: Early CD group
AG: Advanced CD group
CG: Healthy controls group
rDNA: Ribosomal DNA
CDAI: Disease Activity Index of CD
PCoA: Principal component analysis
PICRUSt: Phylogenetic investigation of communities by reconstruction of unobserved states
FC: Fecal calprotectin
CRP: C-reactive protein
ASCA: Anti-saccharomyces cerevisiae antibody
p-ANCA: Perinuclear antineutrophil cytoplasmic antibody
ESR: Erythrocyte sedimentation rate
KEGG: Kyoto Encyclopedia of Genes and Genomes database
KOs: KEGG orthologues
SCFA: Short-chain fatty acid
SD: Standard deviation
DSS: Dextran sodium sulfate
