# Supplementary material for: Gut microbiota in the early stage of Crohn’s disease has unique characteristics
Source: Gut Pathog. 2022 Dec 14;14:46. doi: 10.1186/s13099-022-00521-0 (PMC9753350; doi:10.1186/s13099-022-00521-0)
Supplement: Supplementary file 1 — Additional file 1: Figure S1. Sequencing information. A. OTUs Venn diagram analysis; B. Rank abundance curve. The X-axis represents the Rank of OTUs Abundance, and the Y-axis represents the corresponding OTUs Abundance. The Rank-Abundance curve can intuitively reflect the classified Abundance and evenness contained in the sample, that is, in the horizontal direction, the higher the value of the curve on the horizontal axis, the higher the Abundance. In the vertical direction, the flatter the curve, the more uniform the species distribution. Figure S2. Functional predictions of microbiota present in the fecal of CD patients and healthy controls. Significant KEGG pathways of Level 3 for the microbiome of the CD and healthy groups was identified. PICRUSt, Phylogenetic Investigation of Communities by Reconstruction of Unobserved States. [file 13099_2022_521_MOESM1_ESM.docx]

**Gut Microbiota in the Early Stage of Crohn's Disease Has Unique Characteristics**

**Running Title:** Early CD Has Unique Characteristics

Xianzong Ma^1,2#^, Xiaojuan Lu^2#^, Wenyu Zhang^3#^, Lang Yang^2,4^, Dezhi Wang^1,2^, Junfeng Xu^4^, Yan Jia^2^, Xin Wang^2^, Hui Xie^2^, Shu Li^2^, Mingjie Zhang^2^, Yuqi He^2*^, Peng Jin^2,4*^, Jianqiu Sheng^1,2,4*^

^1^Medical School of Chinese PLA, Beijing, 100853, China;

^2^Department of Gastroenterology, The Seventh Medical Center of Chinese PLA General Hospital, Beijing, 100700, China;

^3^Capital medical university, Beijing, 100069, China;

^4^Senior Department of Gastroenterology, The First Medical Center of Chinese PLA General Hospital, Beijing, 100069, China.

^#^These authors have contributed equally to this work.

***Corresponding author:**

Professor Jianqiu Sheng, Department of Gastroenterology, the Seventh Medical Center of Chinese PLA General Hospital, No.5 Nanmencang, Beijing,100700, China. Tel: +86-10-66721299; Fax: +86-10-66721024, Email: shengjianqiu@301hospital.com.cn.

Professor Peng Jin, Senior Department of Gastroenterology, the First Medical Center of Chinese NPLA General Hospital, No.28 Fuxing Road, Beijing, 100853, China. Tel: +86-10-66721299; Fax: +86-10-66721024, Email: jinpeng@301hospital.com.cn.

Professor Yuqi He, Department of Gastroenterology, the Seventh Medical Center of Chinese PLA General Hospital, No.5 Nanmencang, Beijing,100700, China. Tel: +86-10-66721299; Fax: +86-10-66721024, Email: endohe@163.com

**
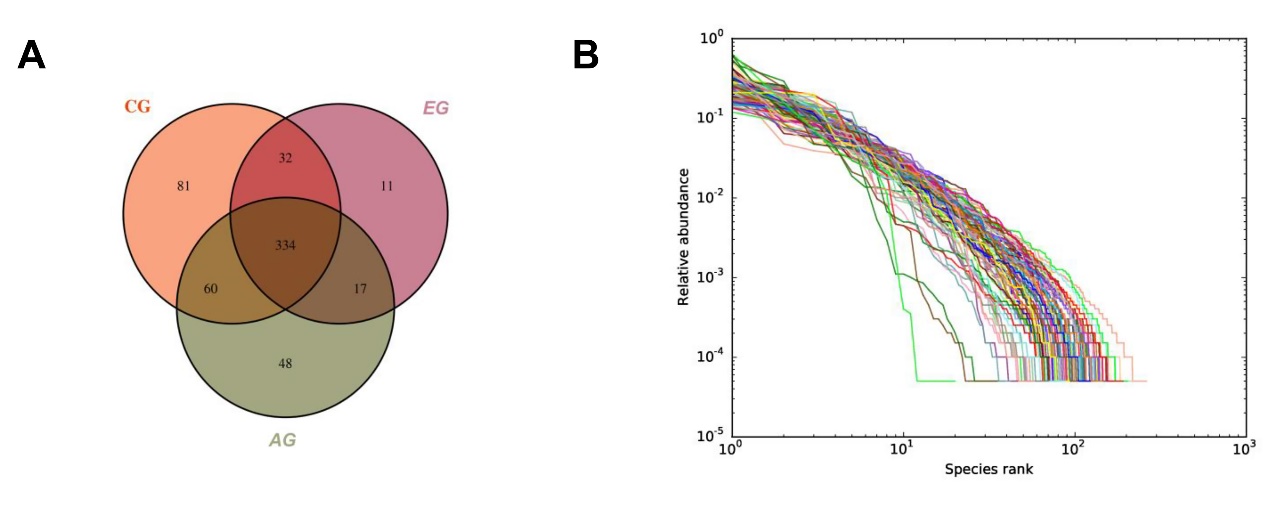
**

**Figure S1.** Sequencing Information. A. OTUs Venn diagram analysis; B. Rank abundance curve. The X-axis represents the Rank of OTUs Abundance, and the Y-axis represents the corresponding OTUs Abundance. The Rank-Abundance curve can intuitively reflect the classified Abundance and evenness contained in the sample, that is, in the horizontal direction, the higher the value of the curve on the horizontal axis, the higher the Abundance. In the vertical direction, the flatter the curve, the more uniform the species distribution.


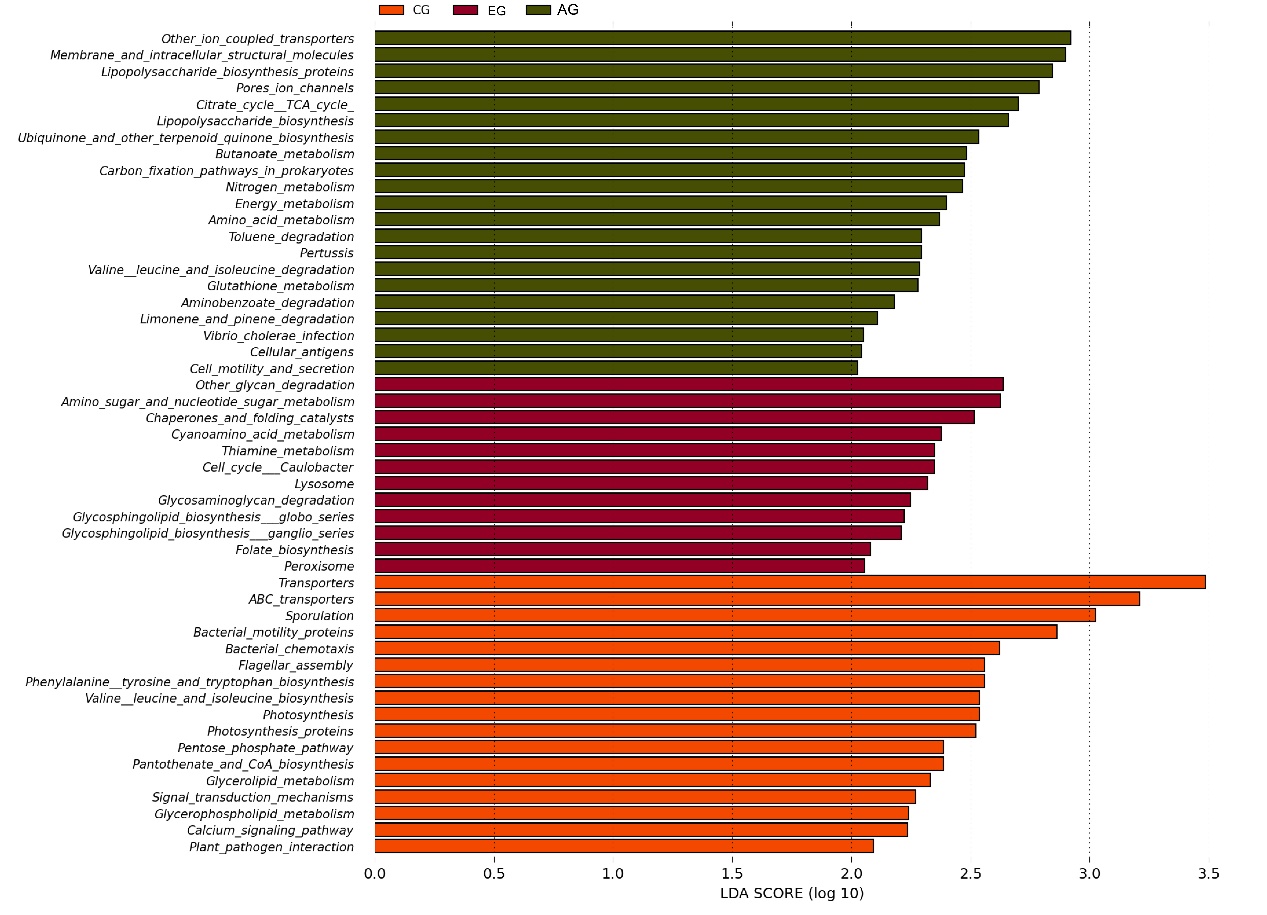


**Figure S2.** Functional predictions of microbiota present in the fecal of CD patients and healthy controls. Significant KEGG pathways of Level 3 for the microbiome of the CD and healthy groups was identified. PICRUSt, Phylogenetic Investigation of Communities by Reconstruction of Unobserved States.
